# Application of a functionalized ionic liquid extractant tributylmethylammonium dibutyldiglycolamate ([A336][BDGA]) in light rare earth extraction and separation

**DOI:** 10.1371/journal.pone.0201405

**Published:** 2018-08-23

**Authors:** Lina Qiu, Yuhong Pan, Weiwei Zhang, Aijun Gong

**Affiliations:** 1 School of Chemistry and Biological Engineering, University of Science and Technology Beijing, Beijing, China; 2 Beijing Key Laboratory for Science and Application of Functional Molecular and Crystalline Materials, University of Science and Technology Beijing, Beijing, China; 3 Basic Experimental Center for Natural Science, University of Science and Technology Beijing, Beijing, China; University of Edinburgh, UNITED KINGDOM

## Abstract

A type of functionalized ionic liquid extractant, tributylmethylammonium dibutyldiglycolamate ([A336][BDGA]), was synthesized, and its extraction ability of light rare earth elements, namely, La(III), Ce(III), Pr(III) and Nd(III), wascompared with that of other functionalized ionic liquid extractants, including tributylmethylammonium dioctyldiglycolamate and diglycolic amide extractants N,N,N,N-tetrabutyl-3-oxapentane-diamide (TBDGA) and N,N,N,N-tetraoctyl-3-oxapentane-diamide (TODGA). The study of extraction behavior indicated that the ionic liquids exhibited better extraction properties than the amides under lower acidity conditions. The effects of the pH and concentration of the extractant on the extraction behavior of [A336][BDGA] on light rare earths were determined. A separation strategy of mixed light rare earths was investigated by means of extraction chromatography using [A336][BDGA] as the stationary phase. As a result, nearly pure La(III), Ce(III), Pr(III) and Nd(III) were obtained, respectively. A new strategy of separating mixed light rare earths was established by means of extraction chromatography. The stationary phase of [A336][BDGA] was used in this strategy. The four rare earths elements, La(III), Ce(III), Pr(III) and Nd(III), achieved baseline separation.

## Introduction

The rare earths (REs), which comprise scandium, yttrium and the lanthanide metallic elements, are widely used in not only traditional industries but also modern material industries, since they show excellent luminescence, electronic, magnetic and thermological properties. To obtain their best properties, high purity is necessary in most cases. However, due to their similar chemical and physical properties, the separation of rare earths has been a challenge for decades. Among all the separation methods, solvent extraction is considered to be the most desirable [1] because this technique has greater processing capacity, a faster reaction rate, a better separation effect in comparison with others, such as crystallization, precipitation and redox processes, and so on. In addition, the use of simple devices and its low cost make it more applicable in industrial production [2].

As the foundation solvent extraction, finding a proper extractant has attracted more and more attention in recent years. Up to now, manifold extractants have been synthesized and developed, which contain organophosphorous extractants, carboxylic acid extractants, amine extractants, amide extractants and so on. Among the above extractants, organophorous extractants are the most widely used [3], but they are also toxic and harmful to the environment. Conversely, amides are the most popular at research level, owing to their merits of decomposing products sufficiently and bringing no secondary pollution [4–8]. Although they have attracted significant interest from researchers, most were applied to high-level liquid waste and nuclear fuel recycling [9–13], with applications in rare earth separation rarely reported.

Ionic liquids are ‘designer solvents’ composed entirely of ions. Their physicochemical properties are controllable by changing discrete cations and anions [14]. With brilliant technical potential, tunable compounds can be developed as functionalized ionic liquids containing certain functional groups. Most results have been obtained by using ionic liquids as diluents [15–17], while during the exploration of the extraction of rare earths, more and more work has been reported on designing ionic liquids as highly efficient extractants [18–25].

Regarded as a versatile and affordable cation source for the synthesis of a new family of hydrophobic ionic liquids, Aliquat-336 can be combined with acidic extractants such as organophosphorous extractants, carboxylic acid extractants and so on by a simple replacement reaction. Mikkola et al. [20] synthesized strongly hydrophobic ionic liquids containing the A336 cation, whose density was lower than 0.9 g/mL, which is highly desirable for counter-current extraction. Considering its unique advantages, several Aliquat-336-based ionic liquids were prepared and exploited for various applications. Sun et al. and Yang et al. [21–23] studied the extraction behaviors of rare earths by an A336 quaternary ammonium salt ionic liquid in aqueous solution. Their results showed that inner synergism plays a dominant role when these functionalized ionic liquids a used as extractants by comparing them with those of a mixture of their corresponding precursors. Guo et al. [24] synthesized [A336]^+^ [P204]^−^and [A336]^+^ [P507]^–^. A study of separation factors (β) indicated that [A336]^+^ [P204]^−^and [A336]^+^[P507]^−^could be suitable for the separation of heavy rare earths in a nitrate medium and the separation of light rare earths in a chloride medium. Rout et al. [18,25] developed two kinds of ionic liquids, [A336]^+^[DEHP]^−^and [A336]^+^ [DGA]^–^, and studied the extraction behaviors of Eu(III) and Am(III) from aqueous solutions. The results showed that [A336]^+^ [DGA]^−^was a potential candidate for the separation of Eu(III) from Am(III).

**Fig 1:**
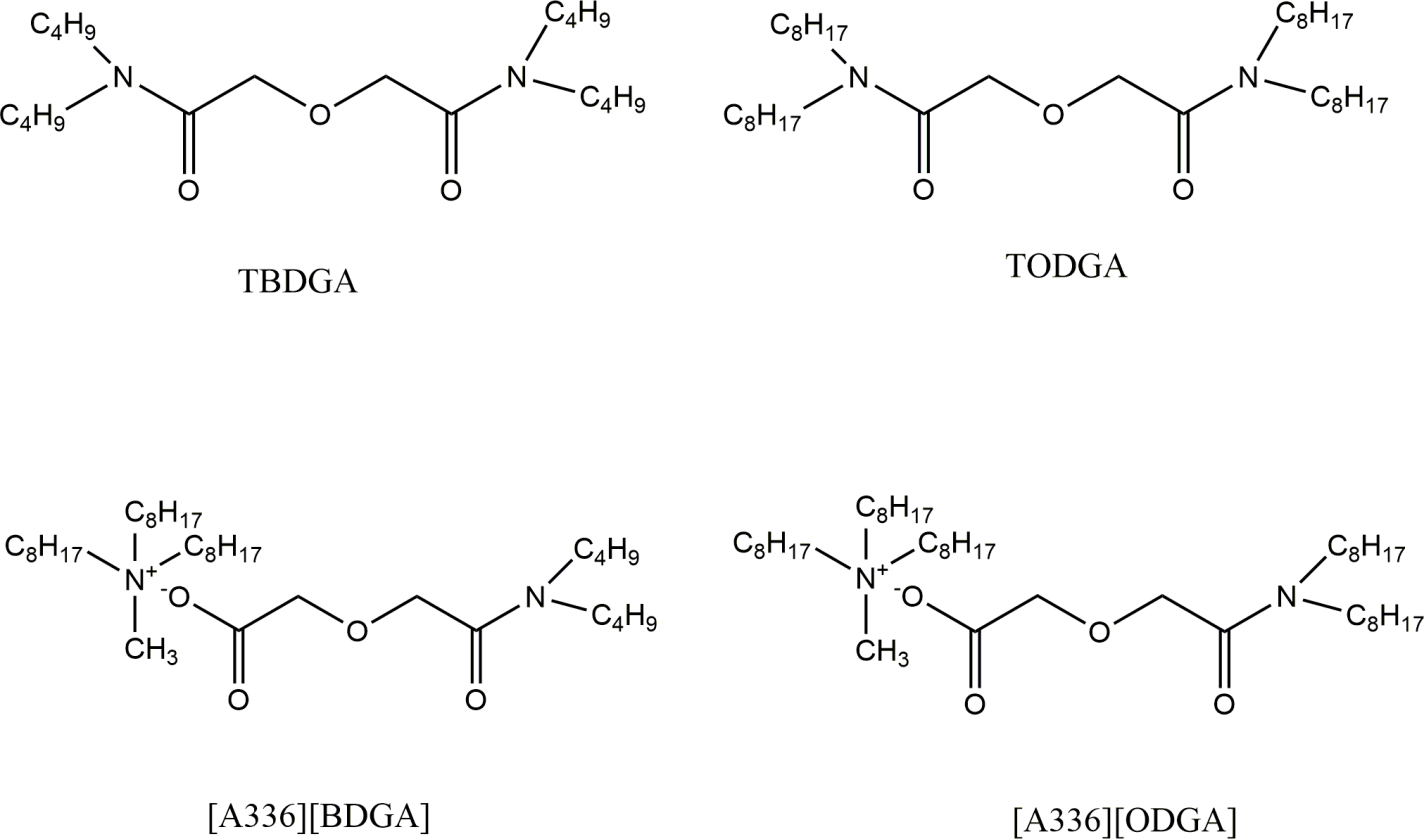
Chemical structures of TBDGA, TODGA, [A336][BDGA] and [A336][ODGA] extractants.

In the study, a kind of quaternary ammonium ionic extractant, [A336][BDGA], is synthesized. The obtained extractant of [A336][BDGA] is taken as the stationary phase to separate mixed rare earths, La(III), Ce(III), Pr(III) and Nd(III). The extraction properties of four kinds of extractants, [A336][BDGA], [A336][ODGA], TBDGA and TODGA were also compared, whose structures are showed in Fig 1. The extraction behaviors of the functionalized ionic liquid [A336][BDGA] on light rare earths were investigated to make a preliminary inference of the extraction mechanism. The studies involved optimization of the chromatography parameters, including the acidity of the eluent, the amount of stationary phase and the diameter of the column. Finally, nearly pure La(III), Ce(III), Pr(III) and Nd(III) were obtained, respectively under the optimized experimental conditions.

## Materials and methods

### Materials and reagents

All of the chemicals and reagents used in this study were of analytical grade. Nitric acid, dichloromethane, Aliquat 336, sodium nitrate, diglycolic anhydride, n-dioctylamine, n-dibutylamine and sodium hydroxide were purchased from Sinopharm Chemical Reagent Co., Ltd. and used as received. The triethylamine was freshly distilled from calcium hydride before use. Highly pure oxides of lanthanum, cerium, praseodymium, neodymium and other rare earths were kindly provided by the Baotou Research Institute of Rare Earths. The rare earth nitrates were prepared by dissolving the pure oxides into nitric acid and then diluting to a certain volume by deionized water. Aliquat 336 is considered here as trioctylmethylammonium chloride or tricaprylmethylammonium chloride. Chromosorb-W (dimethyl dichlorosilane- treated acid washed celite diatomaceous silica, mesh size 200–300) was procured from Sinopharm Chemical Reagent Co., Ltd., washed with distilled water and alcohol followed by vacuum drying before use.

### Synthesis of four extractants

The ionic liquids [A336][BDGA] was prepared by an acid/base neutralization reaction combining [A336][OH] and HBDGA. [A336][OH] was first synthesized by the procedure described elsewhere [22]. The mono-substituted oxa-pentaneamide HBDGA was synthesized by a modified method: 50 g (0.373mol) of diglycolic acid was dissolved in 140 mL of acetic anhydride and drops of phosphorus acid were added as an initiating agent. The temperature rose to the boiling point of acetic anhydride (139°C) and the reaction mixture was stirred for 1 h to obtain diglycolicangydride. Acetic anhydride was partially evaporated under reduced pressure distillation at 85°C. The residue was recrystallized from toluene to give the diglycolic anhydride as a white solid. An amounts of 58.5 g (0.504 mol) of diglycolic anhydride was dissolved in 450 mL of 1,4-dioxane and 85.5 mL (0.505 mol) of dibutyl amine, and 40.5 mL (0.502 mol) pyridine was added in dropwise under ice-water bath conditions. Then, the reaction mixture was stirred for 3 h. The 1,4-dioxane was distilled and the crystal appeared when 1:1 hydrochloric acid was dropped into the residual solution. The HBDGA crystal was separated by filtration. The last step to obtain the ionic liquids involved refluxing the [A336][OH] with the mono-substituted oxa-pentane amide in a 1: 1 molor ratio at 60°C for 10 h. The lower aqueous phase was separated and the ionic liquid phase (upper phase) was dried in a rotary evaporator at 70°C for 5 h. A nearly quantitative yield was obtained. The ionic liquid production was characterized by FT-IR and ^1^H NMR. FT-IR (ν/cm^−1^): 2955–2857 (CH_2_), 1733 (-CO in -COO-), 1645 (-CO in -CON), 1466 (-CH_2_-CO), 1217 (C-N) and 1126 (C-O ether linkage). ^1^H NMR (300 MHz, CDCl_3_, Me_4_Si, δ): 4.31 (s, 2H), 4.08 (s, 2H),3.33 (m, 3H), 3.23 (m, 6H), 3.15 (m, 4H), 1.62 (m, 6H), 1.47 (m, 4H), 1.23 (m, 4H) and 0.86(m, 6H).

The other three kinds of extractants, [A336][ODGA], TBDGA and TODGA were synthesized by the relevant methods describe elsewhere [6,18]. The characterization of the products is shown as follows.

**TBDGA:** FT-IR (ν/cm^−1^): 2957–2873 (CH_2_), 1647 (-CO in -CON), 1466 (-CH_2_-CO), 1217 (C-N) and 1121 (C-O ether linkage). ^1^H NMR (300 MHz, CDCl_3_, Me_4_Si, δ): 4.26 (s, 4H), 3.26 (t, 4H), 3.14 (t, 4H), 1.46 (m, 8H), 1.26 (br, 8H) and 0.88 (t, 16H).
**TODGA:** FT-IR (ν/cm^−1^): 2957–2873 (CH_2_), 1647 (-CO in -CON), 1466 (-CH_2_-CO), 1217 (C-N) and 1121 (C-O ether linkage). ^1^H NMR (300 MHz, CDCl_3_, Me_4_Si, δ): 4.27 (s, 4H), 3.26 (t, 4H), 3.14 (t, 4H), 1.49 (m, 8H), 1.24 (br, 40H) and 0.88 (t,16H).
**[A336][ODGA]:** FT-IR (ν/cm^−1^): 2955–2857 (CH_2_), 1733 (-CO in -COO-), 1645 (-CO in -CON), 1466 (-CH_2_-CO), 1217 (C-N) and 1126 (C-O ether linkage). ^1^H NMR (300 MHz, CDCl_3_, Me_4_Si, δ): 4.31 (s, 2H), 4.12 (s, 2H), 3.62 (m, 3H), 3.33 (m, 6H), 3.10 (m, 4H), 1.64 (m, 6H), 1.51 (m, 4H), 1.25 (br, 20H) and 0.86(m, 6H).

### Extraction procedure

The extraction studies were carried out in centrifuge tubes of 10 mL at 298 K. A volume of 2.0 mL of the organic phase and 2.0 mL of the aqueous phase were mixed and shook for 60 min in a desktop constant temperature oscillator to ensure complete equilibration. The mixture was then centrifuged for 3 min at 3000 rpm to enhance phase separation. The concentration of Res(III) in aqueous phase was determined by a V-1000 UV-Visible spectrophotometer and the concentration of REs(III) in the organic phase was calculated by a mass balance. The extraction efficiency (E), distribution ratio (D), separation factor (β) and stripping ratio (St) were calculated by:

$$E\left(\%\right)=\frac{C_{i}-C_{a}}{C_{i}}\times 100\%$$ (1)

$$D=\frac{C_{i}-C_{a}}{C_{a}}$$ (2)

$$St\left(\%\right)=\frac{C_{a}}{C_{i}-C_{a}}\times 100\%$$ (3)

where C_i_ and C_a_ are the initial and final concentrations of REs(III) in the aqueous phase, respectively, (C_i_—C_a_) is the final concentrations of REs(III) in the organic phase and D_1_ and D_2_ are the distribution ratios of REs(III) 1 and 2, respectively. C_a_ is the equilibrium concentration of REs(III) in the aqueous phase and (C_i_—C_a_) is the initial concentration of REs(III) in the organic phase. The experiments were conducted three times parallel, and the relative error between duplicates was less than 5%. The results were reported as mean values.

### Preparation of chromatographic resin material

The extraction chromatographic resin material was prepared by impregnating [A336][BDGA] on Chromosorb-W. A certain amount of [A336][BDGA] was diluted in ethanol (1:2) and was mixed with Chromosorb-W (1:2) and then equilibrated for 24 h in a mechanical shaker. The solvent of the suspension was removed by rotary evaporation. The resultant material was vacuum dried to constant weight. The impregnation ratio of the extractant loaded with the resin was calculated from the difference in the weight of the solid support before and after impregnation of the extractant and was found to be 99%. The mixed RE sample was prepared by dipping the ionic liquid [A336][BDGA] extracted equal La(III), Ce(III), Pr(III) and Nd(III) on the Chromosorb-W and then treating with the procedure described as the extraction chromatographic resin material.

### Column studies

The extraction column was prepared by slurry packing a certain mass of chromatographic resin material in a glass chromatographic column of 15 mm diameter. A 0.5 g mixed rare earths sample was packed after the extraction column was eluted by volumes of certain concentrations of HNO_3_ to reach an equilibrated acidity. After that, the HNO_3_ solution eluted the column with a constant flow rate of 1.0 mL/min with the help of pressure infusion and the effluent was picked up every 2.0 mL, where after the concentration of the rare earths was determined by the method discussed for the extraction procedure. The breakthrough curves were obtained and analyzed.

## Results and discussion

### Comparison of extraction ability of extractants

To compare the extraction ability of the diglycolamide extractants and the ionic liquid extractants, two kinds of diglycolamide extractants, TBDGA and TODGA, as well as two kinds of ionic liquid extractants, [A336][BDGA] and [A336][ODGA], were synthesized and their extraction behaviors for La(III), Ce(III), Pr(III) and Nd(III) in both high acidity and low acidity were investigated. As shown in Table 1, the diglycolamide extractants exhibited high distribution ratios under high acidity, while the ionic liquids showed almost no extraction ability. On the contrary, the results indicated that the distribution ratios of the ionic liquids were much higher than those of the diglycolamide extractants under low acidity in Table 2. In addition, by combining the results in both Tables 1 and 2, it was found that when the acidity varied from high acidity to low acidity, the distribution ratios of the diglycolamide extractants decreased. However, those of the ionic liquids increased conversely. It also cannot be ignored that the butyl groups showed a positive effect on the extraction ability in both diglycolamide and ionic liquids extractants. Therefore, significant research has been focused on the excellent extraction ability of TODGA, and not only investigating its extraction behaviors, but also developing its applications, such as supplied liquid membranes and extraction chromatography. Meanwhile, there were also studies showing that TBDGA performs better than TODGA in trivalent lanthanide extractions, which agrees with our experimental results. In general, the decreased incomplexing ability of diglycolamides with long alkyl chains to rare earths could be explained by steric hindrance caused by bulky substituents [5,26,27].

**Table 1:** Distribution ratios of [A336][BDGA], [A336][ODGA], TBDGA and TODGA extractants in high acidity.

Extractants	D_La_	D_Ce_	D_Pr_	D_Nd_
[A336][BDGA]	0.30	0.44	0.81	0.99
[A336][ODGA]	0.25	0.39	0.75	0.97
TBDGA	26.64	45.47	46.74	94.78
TODGA	7.36	15.05	25.52	77.36

([[A336][BDGA]] = 0.25 mol/L, [RE(III)]_initial_ = 0.04 mol/L, [NaNO_3_]_initial_ = 1.0 mol/L and pH_e_ = 0.10).

**Table 2:** Distribution ratios of [A336][BDGA], [A336][ODGA], TBDGA and TODGA extractants in low acidity.

Extractants	D_La_	D_Ce_	D_Pr_	D_Nd_
[A336][BDGA]	212.00	474.48	479.51	1716.08
[A336][ODGA]	176.50	191.14	219.25	239.24
TBDGA	14.21	25.69	58.44	77.82
TODGA	5.02	10.31	18.02	27.42

([[A336][BDGA]] = 0.25 mol/L, [RE(III)]_initial_ = 0.04 mol/L, [NaNO_3_]_initial_ = 1.0 mol/L and pH_e_ = 1.25).

### Effect of [A336][BDGA] concentration

**Fig 2:**
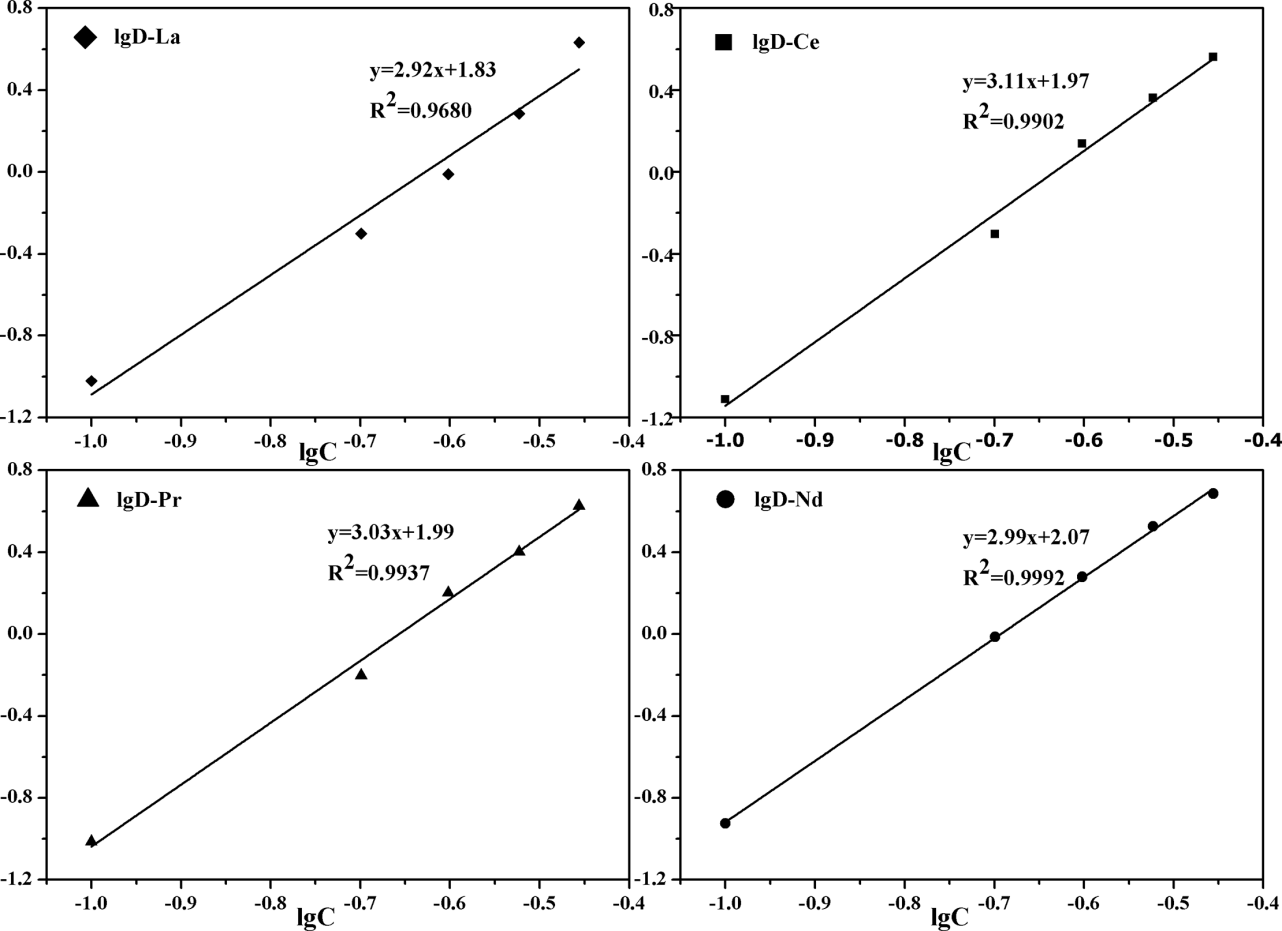
Effect of the concentration of [A336][BDGA]. **The date of the [A336][BDGA] concentration can be seen in S1 File.** ([RE(III)]_initial_ = 0.04 mol/L, [NaNO_3_]_initial_ = 1.0mol/L and pH_e_ = 0.55).

Since both the cation [A336]^+^ and the anion [BDGA]^-^ complex with RE(III), an ion association mechanism can be achieved. In order to explain the mechanism clearly, the effect of the concentration of [A336][BDGA] was investigated. As shown in Fig 2, the distribution ratios of La(III), Ce(III), Pr(III) and Nd(III) increase with an increase in the concentration of the ionic liquid [A336][BDGA]. Through the linear regression analyses of the extraction data, four straight lines with slopes of 2.92, 3.11, 3.03 and 2.99 were calculated, respectively, which indicated the involvement of three molecule of [A336][BDGA] during the extraction process. Therefore, a proposed mechanism for the extraction of light RE(III) by [A336][BDGA] dissolved in molecular dilute may be:

$$RE_{\left(aq\right)}^{3+}+3NO_{3}^{-}{}_{\left(aq\right)}+3[A\;336]{\left[BDGA\right]}_{\left(org\right)}⇄RE{\left(NO_{3}\right)}_{3}\cdot 3[A\;336]{\left[BDGA\right]}_{\left(org\right)}$$ (4)

### Effect of acidity

**Fig 3:**
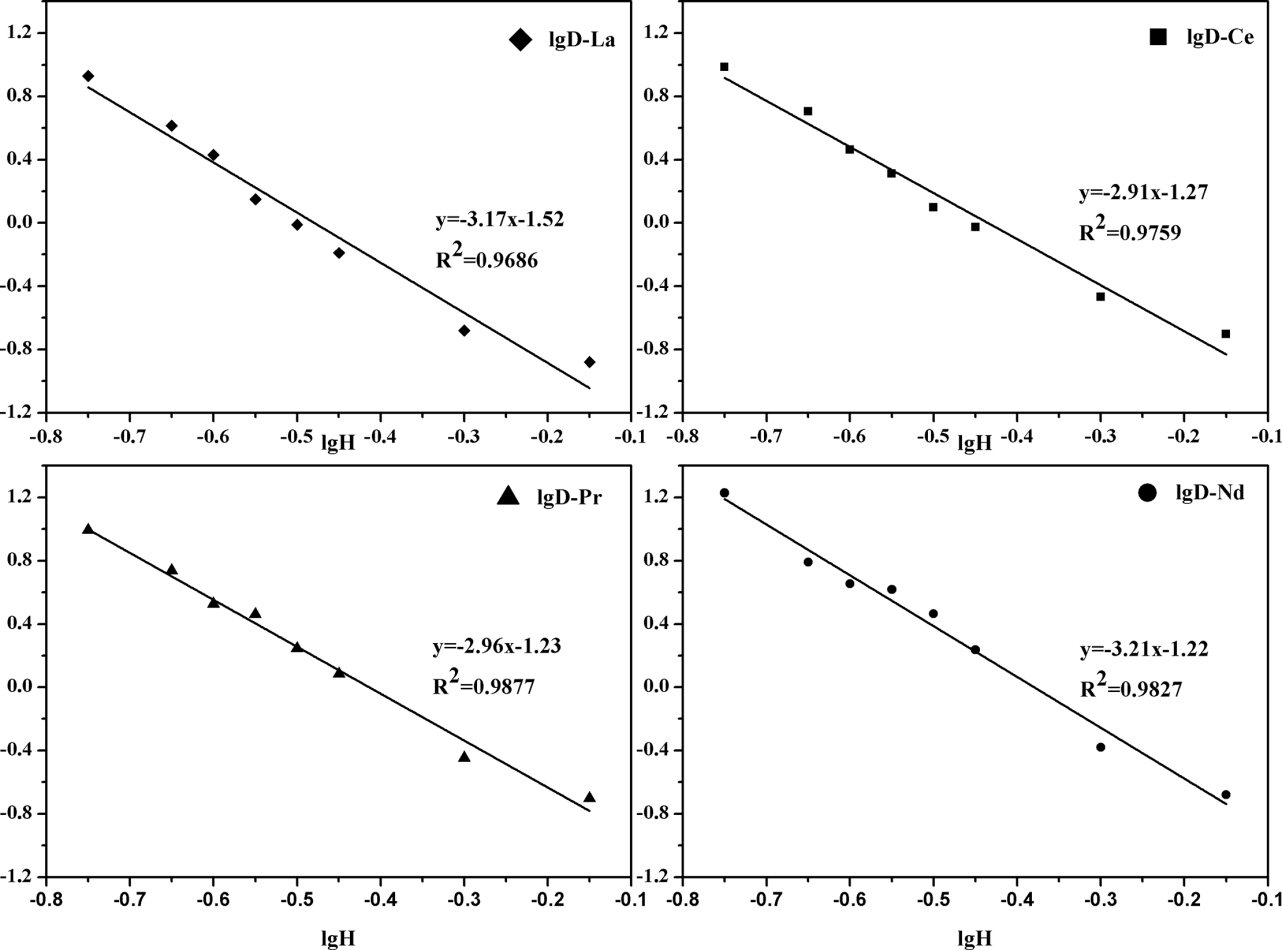
Effect of acidity. **The date of the acidity can be seen in S1 File.** ([[A336][BDGA]] = 0.25 mol/L, [RE(III)]_initial_ = 0.04 mol/L and [NaNO_3_]_initial_ = 1.0mol/L).

Fig 3 shows that the acidity has a negative effect on rare earth extraction. This result was in agreement with the results from Tables 1 and 2. As showed in Fig 3, linear regression analyses of the extraction data resulted in four straight lines with slopes of -3.17, -2.91, -2.96 and -3.21, respectively, which suggested the release of three molecule of H^+^ during the extraction process. Therefore, the mechanism for the extraction of light RE(III) by [A336][BDGA] dissolved in molecular dilute can be inferred from the following equation:

$$RE^{3+}+3\left[A336\right]\left[BDGA\right]\cdot HNO_{3}↔RE{\left(NO_{3}\right)}_{3}\cdot 3\left[A336\right]\left[BDGA\right]+3H^{+}$$ (5)

### Extraction of other Ln(III) from nitric acid medium

**Fig 4:**
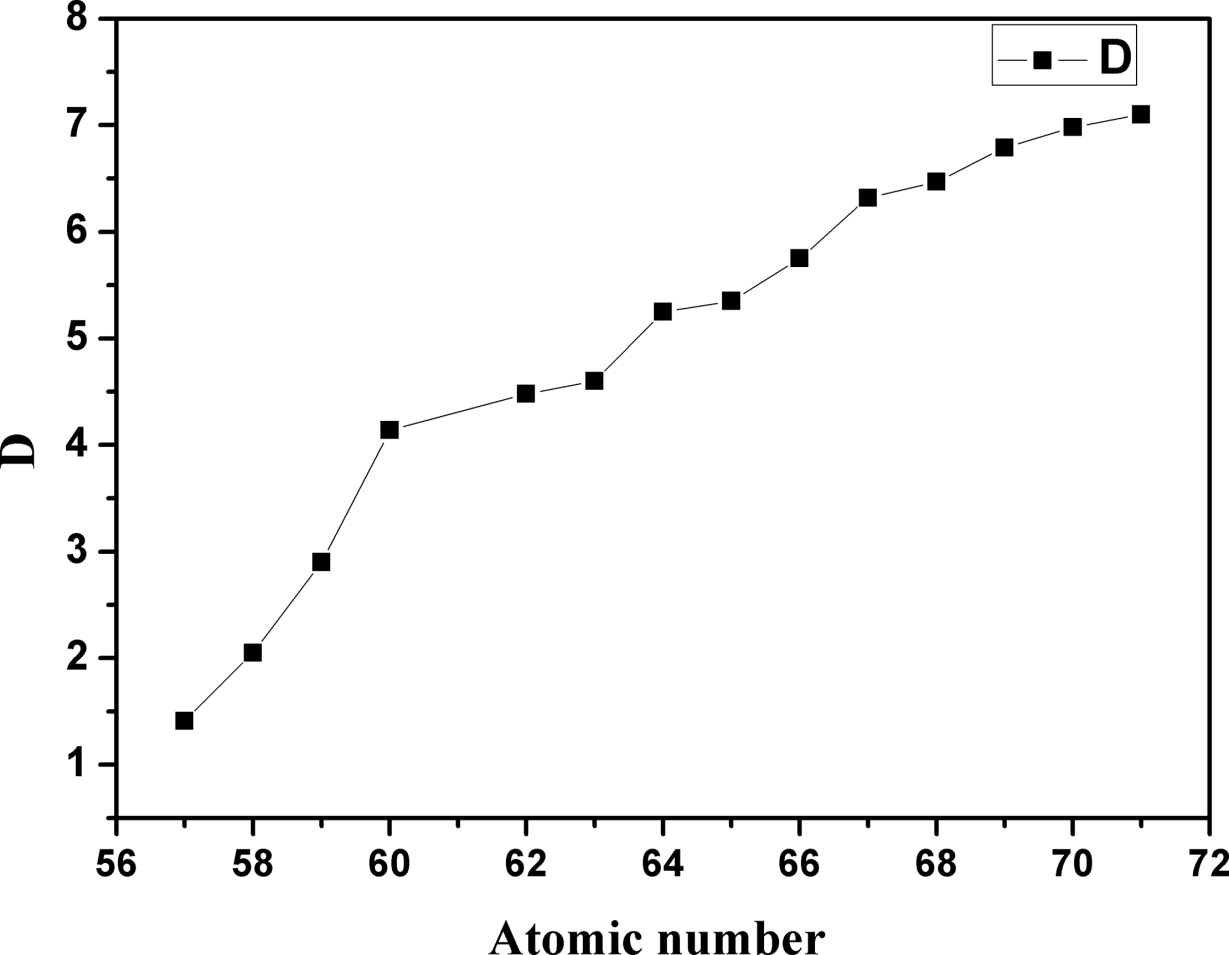
Relationship between distribution ratio (D) and atomic number of Ln(III). **The date of the distribution ratio can be seen in S1 File.** ([[A336][BDGA]] = 0.25 mol/L, [RE(III)]_initial_ = 0.04 mol/L, [NaNO_3_]_initial_ = 1.0 mol/L and pH_e_ = 1.25).

The relationship between the distribution ratios of the trivalent lanthanides with the ionic liquid extractant [A336][BDGA] in a nitric acid medium and their atomic numbers were also investigated, with the results provided in Fig 4. It was found that the distribution ratios of the trivalent lanthanides increase with increasing atomic number, which is in agreement with those previous reports for the extraction of lanthanides using diglycolamides, like N,N,N’,N’-tetra-octyldiglycolamide or N,N’-dimethyl-N,N’-diphenyldiglycolamide, and quarter ammonium ionic liquids, such as [A336][P507] and [A336][P204], but was different from those reported for the extraction of Ln(III) from nitrate solutions with malonamides by Mowafy et al. The hydration energy of the Ln(III) ions and the degree of the basicity of the extractant to dehydrate Ln(III) ion may play an important role in extraction [21].

As shown in Fig 4, the sequence of Ln(III) extracted by [A336][BDGA] in nitric acid tends to be smooth after Nd(III), which suggested that the selectivity of light rare earths separation was higher than that of the heavy rare earths. On the contrary, as reported by Guo et al. the distribution ratios decreased with increasing atomic number of Ln(III) when the A336 was used alone as the extractants in a nitric medium. When the ionic liquid [A336][BDGA] was used in Ln(III) separation, both the cation [A336]^+^ and the anion [BDGA]^-^ combined with the Ln(III) formed the larger extracted complex. The difference of distribution ratios between the A336 and the [A336][BDGA] may be due to the inner synergistic effect between the extractant cation and anion to Ln(III). Alternatively, the extraction sequence of Ln(III) was also attributed to its ionic radius. This result can be related to the hydration energy of the Ln(III) ion during extraction [21,28].

### Stripping properties

**Fig 5:**
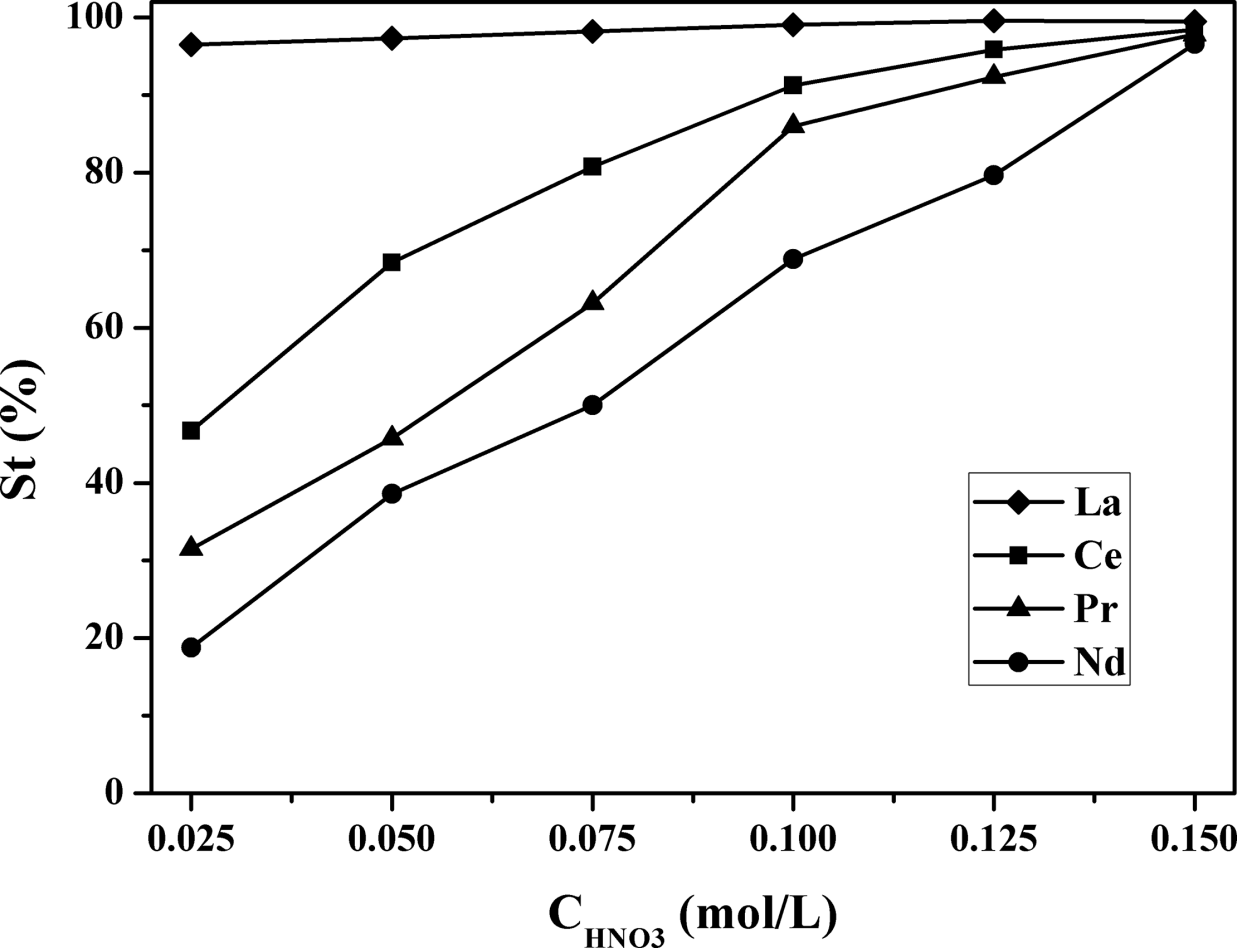
Stripping of RE(III) with HNO_3_ from loaded organic phase. **The date of the HNO**_**3**_ **concentration can be seen in S1 File.** ([[A336][BDGA]] = 0.25 mol/L, [RE(III)]_initial_ = 0.04 mol/L and [NaNO_3_]_initial_ = 1.0 mol/L).

Stripping experiments of extractant loaded RE(III) in a nitrate medium were investigated. As shown in Fig 5, with increasing HNO_3_ concentration from 0.053 to 0.128 mol/L, the stripping ratios of RE(III) increased correspondingly. It was found that when the HNO_3_ concentration was lower than 0.103 mol/L, the difference of the stripping ratio of each RE(III) was obviously exhibited. However, when the HNO_3_ concentration increased to 0.128 mol/L, the stripping ratios of RE(III) were higher than 90% except the Nd(III) with a value of ~60%. Based on the above analysis, an extractant chromatographic strategy to realize the mixed RE(III) separation was made, considering the acidity as the most important factor.

### Recycling properties

In practical application, the stability and recycling of the extractants are the most important required factors. The stability and recycling of the ionic liquid [A336][BDGA] in a nitric medium was studied by a repeated loading/stripping process. The extractant was recycled three times. The results are showed in Table 3, which indicated that the loss of the extraction efficiency was almost negligible and the extractants could be reused.

**Table 3:** Distribution ratios of [A336][BDGA] extractant in recycling experiment of La(III).

Number of cycle	1	2	3
E_[A336][BDGA]_(%)	87.56	87.43	86.79

([[A336][BDGA]] = 0.25 mol/L, [La(III)]_initial_ = 0.04 mol/L, [NaNO_3_]_initial_ = 1.0 mol/L pH = 1.00)

### Column studies

**Fig 6:**
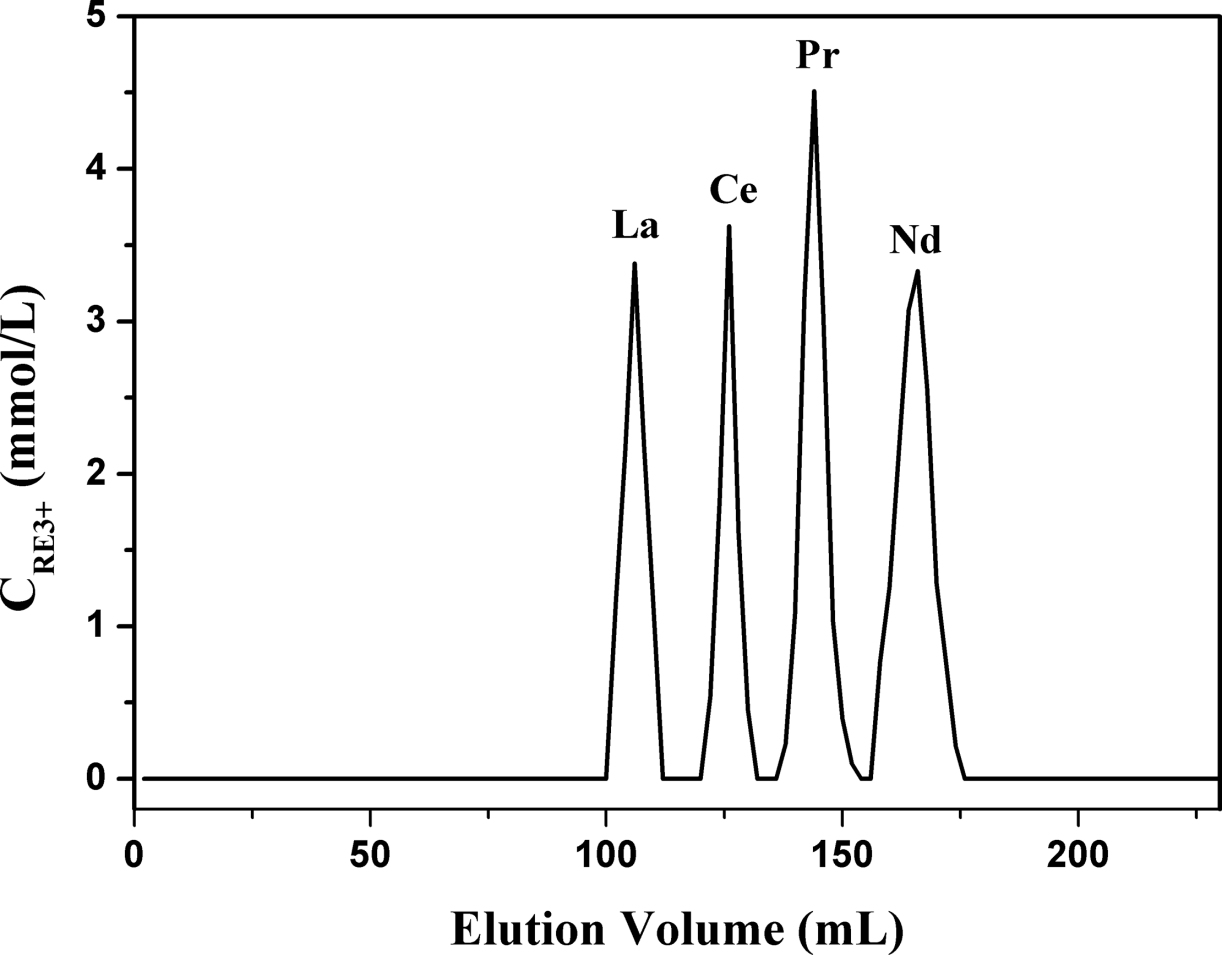
Separation of La(III), Ce(III), Pr(III) and Nd(III) with 0.075 mol/L HNO_3_. The date of the elution volume can be seen in S1 File.

According to the investigation above, an extraction chromate graphic strategy can be explored through using the functionalized ionic liquid [A336][BDGA] as the stationary phase. The acidity of the eluent, the amount of the stationary phase and the diameter of the column were taken into consideration as the experimental conditions to be optimized. The results showed that the acidity could obviously affect the separation of rare earths. The retention time became shorter with increasing acidity of the eluent, which corresponds with the above study of the extraction behavior. However, the best separation condition was obtained with 0.075 mol/L HNO_3_ as the eluent. Additionally, the diameter of the column and the amount of the stationary phase in the column could affect the efficient separation of light rare earths. As shown in Fig 6, nearly pure La(III), Ce(III), Pr(III) and Nd(III) was obtained with a consumption of 0.075 mol/L HNO_3_ less than 220 mL and 100 g of the chromatographic resin material. Therefore, this extraction chromatographic strategy can not only achieve satisfied separation but can also be friendly to the environment because of the low consumption of acid.

## Conclusions

In this study, a functionalized ionic liquid extractant, [A336][BDGA], was synthesized and the comparison of their extraction behaviors on light rare earths among [A336][BDGA], [A336][ODGA], TBDGA and TODGA was conducted. As a result, the ionic liquid extractants showed better extraction ability than the diglycolamide extractants in low acidity and the butyl group performed better in extracting in both diglycolamide and ionic liquid extractants, which suggested that [A336][BDGA] is a good extractant in low acidity. The extraction mechanism was preliminarily inferred by studying its extraction behaviors on the REs(III). It was found that about three molecules of [A336][BDGA] complex with a molecule of RE(III) to form RE(NO_3_)_3_·3[A336][BDGA] during the extraction process and the acidity has a negative influence on the extraction of [A336][BDGA] on RE(III). The separation of light REs(III) could be achieved by means of an extraction chromatographic strategy using [A336][BDGA] as the stationary phase. With 0.075 mol/L HNO3 as the eluent to make light REs(III) form RE^3+^ into the eluent and then achieve the purpose of separation. In addition, the stripping and recycling studies indicated that the ionic liquid extractant [A336][BDGA] could be reused. In conclusion, [A336][BDGA] has the merits of high selectivity and low acid consumption in light rare-earths extraction and separation. This would be a positive factor in industry applications.

## Supporting information

S1 File [A336][BDGA] concentration, acidity, distribution ratio, HNO_3_ concentration and elution volume date of all the figs in this study. (OPJ)
